# Feeding a *Saccharomyces cerevisiae* fermentation product before and during a feed restriction challenge on milk production, plasma biomarkers, and immune function in Holstein cows

**DOI:** 10.1093/jas/skad019

**Published:** 2023-01-14

**Authors:** Danielle N Coleman, Qianming Jiang, Matheus G Lopes, Luciano Ritt, Yusheng Liang, Ahmad Aboragah, Erminio Trevisi, Ilkyu Yoon, Juan J Loor

**Affiliations:** Department of Animal Sciences, Division of Nutritional Sciences, University of Illinois, Urbana, IL 61801, USA; Department of Animal Sciences, Division of Nutritional Sciences, University of Illinois, Urbana, IL 61801, USA; Department of Animal Sciences, Division of Nutritional Sciences, University of Illinois, Urbana, IL 61801, USA; NUPEEC (Núcleo de Pesquisa, Ensino e Extensão em Pecuária), Departamento de Clínicas Veterinária, Programa de Pós-Graduação em Biotecnologia, Universidade Federal de Pelotas, 96010-610, Pelotas, RS, Brazil; Department of Animal Sciences, Division of Nutritional Sciences, University of Illinois, Urbana, IL 61801, USA; Departmento de Zootecnia, Universidade Federal do Rio Grande do Sul, Porto 90040-060, Alegre, RS, Brazil; Department of Animal Sciences, Division of Nutritional Sciences, University of Illinois, Urbana, IL 61801, USA; Department of Animal Sciences, Division of Nutritional Sciences, University of Illinois, Urbana, IL 61801, USA; Department of Animal Sciences, Food and Nutrition (DIANA), Research Center Romeo and Enrica Invernizzi for sustainable dairy production (CREI), Faculty of Agriculture, Food and Environmental Science, Università Cattolica del Sacro Cuore, 29122 Piacenza, Italy; Diamond V, Cedar Rapids, IA 52404, USA; Department of Animal Sciences, Division of Nutritional Sciences, University of Illinois, Urbana, IL 61801, USA

**Keywords:** feed restriction, immune function, lactation, leaky gut

## Abstract

Periods of decreased feed intake may disrupt function of the intestinal barrier. Feeding NutriTek^®^ (NTK; Diamond V, Cedar Rapids, IA), a postbiotic from *S. cerevisiae* fermentation (SCFP), improved health and supported anti-inflammatory functions. We investigated the effects of feeding NTK to cows before and during a period of feed restriction (FR) designed to model periods of intestinal barrier dysfunction. In total, 16 multiparous cows (97.1 ± 7.6 DIM; n = 8/group) were fed a control diet (CON) or CON plus 19 g/d NTK for 9 wk (Phase 1; P1) and then were subjected to an FR challenge for 5 d, during which they were fed 40% of their ad libitum intake from the 7 d prior to FR. Milk yield (MY) and DMI were collected daily. During FR, milk was collected daily for composition, blood daily to measure plasma biomarkers and to measure monocyte and neutrophil phagocytosis and oxidative burst on d 1, 3, and 5. Data were analyzed using a mixed model in SAS 9.4. All data were subjected to repeated measures ANOVA. Dietary treatment (TRT), Day, and their interaction (TRT × Day) were considered as fixed effects and cow as the random effect. For analysis of P1, data collected during a 7-d adaptation phase were used as a covariate. During P1, NTK cows tended to have greater DMI and had greater fat, ECM and FCM yields, and feed efficiency (ECM/DMI and FCM/DMI). Protein yield tended to be greater in NTK compared with CON cows. A tendency for greater monocyte phagocytosis was detected with NTK. However, during FR, feeding NTK led to lower MY and lactose yield and tended to lower solids percentage. While NTK cows tended to have reduced neutrophil oxidative burst than CON cows during FR (NTK: 26.20%, CON: 36.93%), there was no difference in phagocytosis (NTK: 7.92%, CON: 6.31%). Plasma biomarkers of energy metabolism, liver function, inflammation, and oxidative stress during the FR period did not differ. Overall, results suggested that feeding NTK increased the yield of FCM, ECM, feed efficiency and milk components prior to FR.

## Teaser

A *Saccharomyces cerevisiae* fermentation product fed to lactating cows enhances production performance.

## Introduction

Throughout their life, dairy cattle are exposed to a variety of situations where intakes are reduced or restricted, leading to lower performance and altered immunometabolism. The effects of feed restriction (FR) on metabolism (e.g., increased adipose tissue mobilization, reduced milk production) have been well-documented, and work over the last decade has begun to underscore the negative effects of FR on immune function and intestinal barrier function in ruminants (Zhang et al., 2013; Kvidera et al., 2017a, 2017b). During FR, proliferation and migration rates of epithelial cells and villus heights are reduced, which is also associated with changes in the intestinal immune response capacity and the microbiome (Ferraris and Carey, 2000; Hodin et al., 2011). These changes in the gut barrier increase the risk of microbial and endotoxin translocation to the systemic circulation, inducing inflammatory and immune responses by the animal (Kvidera et al., 2017a). One way to mitigate the negative effects of FR on metabolism, gut barrier function, and immune activation may be through nutritional interventions that promote the function of both the intestinal epithelial and immune cells.

Postbiotics from *Saccharomyces cerevisiae* fermentation (SCFP) are a common feed supplement produced via the fermentation of yeast and contain compounds such as amino acids, nucleotides, B vitamins, organic acids, and oligosaccharides (Hristov et al., 2010). While sometimes inconsistent, most studies have reported positive effects of SCFP supplementation on milk yield (MY), DMI, ruminal pH and fermentation, and immune function (Hristov et al., 2010; Poppy et al., 2012; Yuan et al., 2015). Supplementation with SCFP has also generated beneficial health effects during immunological challenges in both dairy cows and calves including the early life and weaning periods (Alugongo et al., 2017; Harris et al., 2017), calf respiratory challenge (Mahmoud et al., 2020), the periparturient period (Knoblock et al., 2019), an aflatoxin challenge (Jiang et al., 2018), and heat stress (Al-Qaisi et al., 2020). Furthermore, SCFP has been associated with reductions in mastitis (Ferguson et al., 2018) and improvements in udder health and barrier function during a subclinical mastitis challenge (Vailati-Riboni et al., 2021). Such improvements in immune responses and barrier function have been linked to the bioactive compounds in SCFP, such as glucans, vitamins, and amino acids, which have been observed to activate immune cells, thereby priming the immune system (Li et al., 2005; Yoshii et al., 2019; Coleman et al., 2020). Thus, SCFP may have beneficial effects on priming immune responses and barrier function and promoting milk production during a period of FR in dairy cows.

The hypothesis of the present study was that supplementation of an SCFP before and during an FR challenge would improve production, health, and support anti-inflammatory functions. Our specific objective was to investigate the effects of SCFP supplementation 9 wk prior to and during a period of 5 d abrupt FR on milk production, immune function, and plasma biomarkers of inflammation, oxidative stress, metabolism, and inflammation.

## Materials and methods

### Animal procedures

All the experimental procedures were approved by the University of Illinois (Urbana-Champaign) Institutional Animal Care and Use Committee (no. 17166). In total, 16 multiparous Holstein cows past peak lactation (>60 DIM) were used in a randomized complete block design conducted from February to April 2020. All cows were housed in a freestall system equipped with individual feeding gates (American Calan Inc., Northwood, NH) and were fed once daily at approximately 0800 hours. Milking occurred two times per day at 0400 and 1530 hours. Cows were blocked by DIM (97.1 ± 7.6 d) and parity (3.4 ± 0.62), MY, BCS, and SCC (Supplemental Table 1). After 1 wk of adaptation, cows were randomly assigned within block to one of two treatments (eight per treatment): 1) a control group receiving the basal TMR with no supplementation (CON) or 2) basal TMR with 19 g/d of a *Saccharomyces cerevisiae* fermentation product (NTK; NutriTek®, Diamon V, Cedar Rapids, IA), top-dressed. This is the commercially recommended dose for the type of animal used in this trial. The ration was formulated to meet NRC (2001) requirements and is presented in Table 1. The diet was formulated for cows at 180 DIM, BW of 703 kg, MY of 35.4 kg/d with a target of 3.50% milk fat and 3.12 milk protein and predicted DMI of 24.9 kg/d. The diet included corn silage, alfalfa hay, canola and corn gluten meal, ground shelled corn, rumen-protected lysine and methionine, rumen-inert fat, rumen-bypass protein, vitamin and mineral mix, and the ionophore Rumensin (Elanco, Greenfield, IN). The experimental period lasted 68 d and was divided into two phases: 1) Phase 1 (P1) from the beginning of the experimental period until d 63, and 2) Phase 2, from d 64 to 68, which represents the FR period. Cows were euthanized at the end of FR for a separate experiment. During Phase 2, cows were restricted to 40% of their ad libitum intake of the previous 5 d. This amount of restriction was chosen based off previous work in which 40% restriction was validated as a method to induce intestinal barrier dysfunction in dairy cattle (Kvidera et al., 2017b).

**Table 1.** Ingredient composition of the diet

Item	Value
Ingredient, % DM	
Corn silage	38.39
Alfalfa hay	17.77
Canola meal	4.93
Corn gluten meal	9.23
Ground shelled corn	21.35
Rumen-inert fat^1^	3.13
Rumen-protected lysine^2^	0.05
Rumen-protected methionine^3^	0.07
Rumen-bypass protein^4^	1.10
Ionophore^5^	0.006
Salt	0.12
Mineral supplement^7^	0.05
Potassium carbonate	0.13
Vitamin-Mineral Mix^6^	3.68
Nutrient Analysis	
CP, %DM	15.89
ADF, %DM	17.28
aNDF, %DM	30.18
NFC, %DM	45.53
Ether Extract, %DM	2.79
Starch, %DM	28.00
NE_L_, Mcal/kg	1.70

^1^ Soy Plus, Landus Cooperative, Ames, IA.

^2^ Ajipro-L-G3, Ajinomoto Heartland Inc., Chicago, IL.

^3^Smatamine M, Adisseo, Alpharetta, GA.

^4^ ProVAAL2 AADvantage, Perdue AgriBusiness, Salisbury, MD.

^5^Rumensin, Elanco, Greenfield, IN.

^6^ Zinpro Availa-Dairy, Zinpro Corporation, Eden Prairie, MN. Contains Zn, Mn, Co, and Cu.

^7^Contained a minimum of 12.5% Ca, 10.4% Na, 2.2% Mg, 8.0% K, 0.1% S, 7.1% Se, 244.5 kIU of vitamin A/kg, 48.9 kIU of vitamin D_3_/kg, and 0.922 kIU of vitamin E/kg.

### Feed and production parameters

Dry matter intake was recorded daily. Individual feed ingredient DM was determined weekly, and the ration was adjusted accordingly to maintain DM ratios of the ingredients. Weekly samples of TMR were frozen at −20 °C and composited monthly for analysis by standard wet chemistry techniques at a commercial laboratory (Dairy One, Ithaca, NY) and used to calculate the consumed diet throughout the experiment.

Body weight and BCS were recorded weekly during Phase 1 and at the start and end of Phase 2. Three individual scorers were used at each time point for BCS and their scores were averaged to avoid bias. Body weight and BCS were evaluated at ~ 1400 hours. Milk production was recorded daily, and samples were collected at each milking two times a week (Tuesdays and Fridays) during Phase 1 and daily during Phase 2. Composite samples were prepared each day in proportion to MY at each milking and were preserved (800 Broad Spectrum Microtabs II; D & F Control Systems Inc., San Ramon, CA), and analyzed for contents of fat, protein, total solids, MUN, and SCC in a commercial laboratory (Dairy One). Based on milk sample analysis, FCM was calculated as follows: 3.5% kg FCM = (0.4324 × kg of MY) + (16.216 × kg of milk fat) (Hutjens, 2010). Energy-corrected milk was calculated as ECM = [12.82 × fat yield (kg)] + [7.13 × protein yield (kg)] + [0.323 × MY (kg)] (Hutjens, 2010). Feed efficiency was calculated as ECM/DMI and FCM/DMI. Energy balance (EB) was calculated weekly during Phase 1 and at the start and end of Phase 2 using NRC equations, as described previously (Moyes et al., 2009).

### Blood collection and analysis

Blood was collected via the coccygeal vein using lithium–heparin vacutainer tubes (BD Vacutainer, BD and CO., Franklin Lakes, NJ) in the morning before feeding on d 1, 30, 63, 64, 65, 66, 67, 68, and 69 of the study. Tubes were placed on ice and transported to the laboratory. For plasma, tubes were centrifuged at 2,500 × *g* at 4 °C for 15 min. Samples were then aliquoted into microcentrifuge tubes and stored at −80 °C until analysis. Plasma non-esterified fatty acids (NEFAs), β-hydroxybutyrate (BHB), glucose, total cholesterol, total bilirubin, creatinine, urea, aspartate aminotransferase (AST), γ-glutamyl transpeptidase (GGT), total plasma reactive oxygen metabolites (ROMs), ferric reducing ability of plasma (FRAP), haptoglobin, ceruloplasmin, paraoxonase (PON) activity, and magnesium (Mg) were analyzed as described previously (Trevisi et al., 2013). Myeloperoxidase (MPO) was measured as described by Bionaz et al. (2007). In addition, blood samples collected on d 1, 30, 64, 66, and 68 were used to analyze monocyte and neutrophil oxidative burst activity and phagocytosis capacity via a flow cytometry-based assay as described previously by Zhou et al. (2018).

### Statistical analysis

Statistical analysis was performed in SAS v9.4 (SAS Institute, Cary, NC) and conducted by phase of the experiment. All data were subjected to repeated measures ANOVA using PROC MIXED. Dietary treatment (TRT), Day, and their interaction (TRT × Day) were considered as fixed effects and cow as the random effect. For analysis of P1, data collected during the adaptation phase were used as a covariate. Block was also included in the model to account for the randomized complete block design. The Kenward–Rogers degrees of freedom approximation was used to determine the denominator degrees of freedom for tests of fixed effects. Covariance structures tested for the repeated measurement were compound symmetry, autoregressive order 1, autoregressive heterogeneous order 1, unstructured, and Toeplitz, and the most-appropriate was chosen for each analysis was used for each analysis based on the corrected Akaike information criterion. The compound symmetry structure was used for P1 MY and DMI, while the autoregressive heterogeneous order 1 was used for other production parameters. The autoregressive order 1 structure was implemented for all FR production parameters, as well as P1 and FR immune function and plasma biomarkers. Normality of the residuals was checked via PROC UNIVARIATE. Somatic cell count data were log-transformed. When TRT × Day interactions were significant the SLICE option was used to separate means. Statistical significance for production and plasma biomarker data was determined at *P* ≤ 0.05, and tendencies at *P* ≤ 0.10. For immune function data, statistical significance was determined at *P* ≤ 0.05, and tendencies at *P* ≤ 0.15.

## Results

### Performance

**Figure 1.**
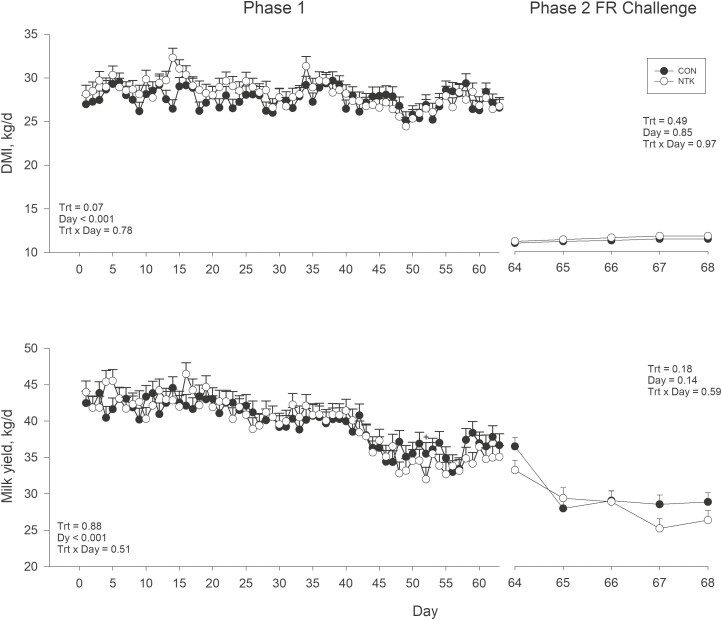
Least square means and associated standard error for DMI and MY of Holstein cows supplemented with a *Saccharomyces cerevisiae* fermentation product (NTK) or a CON for 9 wk and subjected to a FR challenge for 5 d (40% of ad libitum intake). Data are separated according to experimental period; treatment feeding (Phase 1; 0–63 d) and FR challenge (Phase 2; d 64–68). There were eight cows per treatment. *P ≤ 0.05 and +*P* ≤ 0.10.

**Figure 2.**
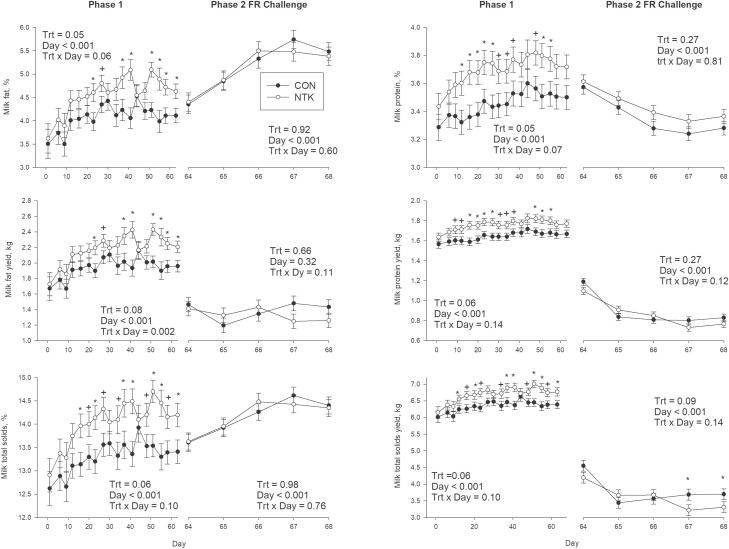
Least squares mean and associated standard error for milk fat percentage and yield, milk protein percentage and milk total solids percentage and yield of Holstein cows supplemented with a *Saccharomyces cerevisiae* fermentation product (NTK) or a CON for 9 wk and subjected to a FR challenge for 5 d (40% of ad libitum intake). Data are separated according to experimental period; treatment feeding (Phase 1; 0–63 d) and FR challenge (Phase 2; d 64–68). Milk composition was measured two times per wk during P1 and daily during FR, and there were eight cows per treatment. *P ≤ 0.05 and +*P* ≤ 0.10.

**Figure 3.**
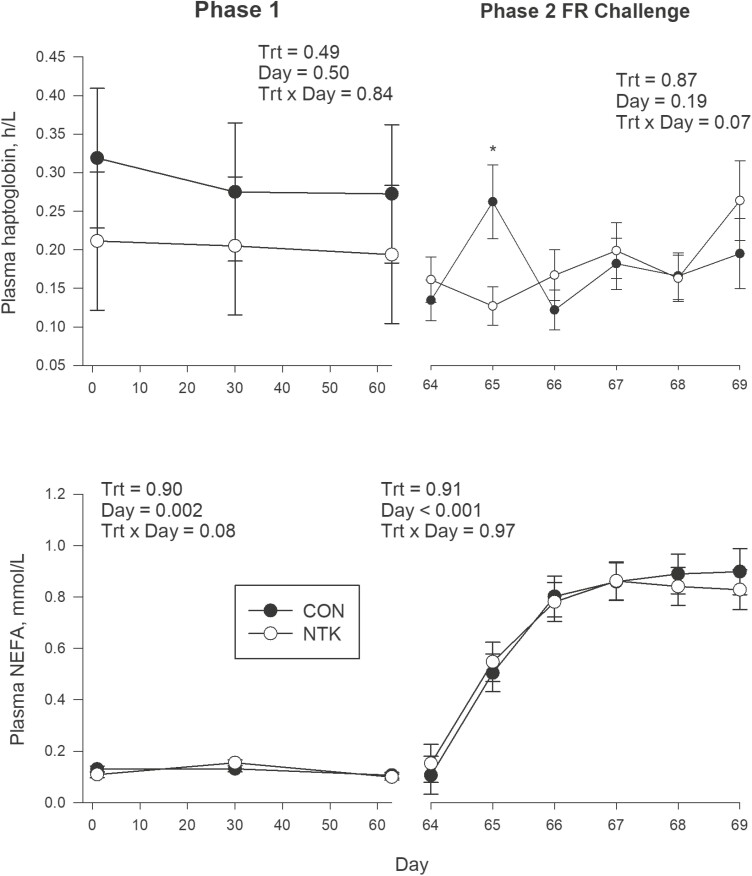
Least square means and associated standard errors for plasma haptoglobin and non-esterified fatty acids (NEFAs) in Holstein cows supplemented with a *Saccharomyces cerevisiae* fermentation product (NTK) or a CON for 9 wk and subjected to a FR challenge for 5 d (40% of ad libitum intake). Data are separated according to experimental period; treatment feeding (Phase 1; 0–63 d) and FR challenge (Phase 2; d 64–68). Blood was collected on ~1 h prior to feeding from the coccygeal vein on d 1, 30, and 63 in Phase 1 and daily during Phase 2. There were eight cows per treatment. *P ≤ 0.05.

During P1, NTK cows tended to have greater DMI than CON (*P* = 0.07; Table 2 and Figure 1), but there was no difference in MY (*P* = 0.88). Fat percentage and yield were greater during P1 with NTK vs. CON (*P* = 0.05 for both), while protein percentage and yield tended to be greater (*P* = 0.08 and 0.06, respectively). There was also a tendency for NTK cows to have greater SNF percentage and yields during P1 (*P* = 0.06 for both). Cows fed NTK had greater MUN (*P* = 0.05). During P1, both ECM and FCM yields were greater with NTK compared to CON (*P* = 0.01 for both). At last, feed efficiency measured as ECM/DMI (*P* = 0.02) and FCM/DMI (*P* = 0.02) was greater in NTK cows during P1. A TRT × Day interaction was observed for milk protein percentage during P1 (*P* = 0.002; Figure 2), with NTK cows having greater protein concentrations on d 12, 16, 20, 23, 27, and 51 (*P* < 0.05) and tending to have greater concentrations than CON cows on d 30, 34, 37, 48, and 55 (*P* < 0.10). There was also a tendency for a TRT × Day interaction detected for P1 fat percentage (*P* = 0.06), fat yield (*P* = 0.07), total solids percentage (*P* = 0.10) and total solids yield (*P* = 0.10). Both fat percentage and yield were greater in NTK cows compared with CON on d 23, 37, 41, 51, 55, 58, and 63 (*P* < 0.05), and tended to be greater on d 27 and 48 (*P* < 0.10). In addition, total solids percentage and yield were greater on d 16, 23, 27, 37, 48, 51, 55, 58, and 63 (*P* < 0.05) and tended to be greater on d 20, 34, and 48 (*P* < 0.10). At last, Day effects were observed for all production parameters during P1 (*P* < 0.05; Figures 2 and 3 and Supplemental Figures S1–4) except for EB, which tended to change across Day (*P* = 0.09). Dry matter intake, MY, and lactose percentage and yield decreased over P1, while ECM, FCM, protein yield, BW, BCS, SCC, and EB and feed efficiency (both ECM/DMI and FCM/DMI) increased.

**Table 2.** Least square means and associated standard error for intake, body weight (BW), body condition score (BCS), EB, MY and milk composition of Holstein cows supplemented with a *Saccharomyces cerevisiae* fermentation product (NTK) or a CON for 9 wk and subjected to a FR challenge for 5 d (40% of ad libitum intake). Data are separated according to experimental period; treatment feeding (Phase 1; 0–63 d) and FR challenge (Phase 2; 64–68).^1^

	Treatment			*P*-value		
Item^2^	CON	NTK	SEM	Trt	Day	Trt × Day
***Phase 1***						
DMI kg/d	27.58	28.15	0.18	0.07	< 0.001	0.78
BW, kg	776	754	18	0.42	< 0.001	0.59
BCS	3.01	2.90	0.09	0.46	0.001	0.28
EB, Mcal/d	18.71	18.36	1.02	0.81	0.09	0.77
MY, kg/d	39.30	39.48	0.65	0.88	< 0.001	0.51
Milk Composition						
Fat, %	4.07	4.57	0.15	0.05	< 0.001	0.06
Fat, kg/d	1.94	2.18	0.07	0.05	< 0.001	0.07
Protein, %	3.45	3.70	0.08	0.08	< 0.001	0.002
Protein, kg/d	1.64	1.76	0.04	0.06	< 0.001	0.14
Lactose, %	4.85	4.83	0.02	0.68	< 0.001	0.19
Lactose, kg/d	2.31	2.30	0.01	0.62	0.001	0.29
Total solids, %	13.30	14.03	0.23	0.06	< 0.001	0.10
Total solids, kg/d	6.34	6.69	0.11	0.06	< 0.001	0.10
MUN^2^, mg/dL	11.40	13.13	0.37	0.05	< 0.001	0.35
SCC^3^	1.61	1.70	0.12	0.62	0.001	0.77
ECM^,4^	49.16	53.31	0.91	0.01	< 0.001	0.21
FCM^5^	48.32	52.54	0.84	0.01	0.001	0.32
ECM/DMI	1.80	1.91	0.03	0.02	0.005	0.72
FCM/DMI	1.77	1.87	0.03	0.02	0.02	0.61
***Phase 2***						
DMI kg/d	11.20	11.46	0.25	0.49	0.85	0.97
BW, kg	758	730	17	0.28	< 0.001	0.76
BCS	2.95	2.81	0.09	0.30	0.01	0.46
EB, Mcal/d	7.79	7.35	1.07	0.77	< 0.001	0.49
MY, kg/d	26.68	24.96	0.54	0.04	< 0.001	0.18
Milk Composition						
Fat, %	5.15	5.13	0.14	0.92	< 0.001	0.60
Fat, kg/d	1.34	1.29	0.05	0.66	0.32	0.11
Protein, %	3.36	3.44	0.04	0.27	< 0.001	0.81
Protein, kg/d	0.89	0.87	0.01	0.27	< 0.001	0.12
Lactose, %	4.72	4.76	0.02	0.37	0.03	0.73
Lactose, kg/d	1.28	1.19	0.02	0.02	< 0.001	0.18
Total solids, %	14.30	14.31	0.13	0.98	< 0.001	0.76
Total solids, kg/d	3.79	3.61	0.07	0.09	< 0.001	0.14
MUN^2^, mg/dL	14.83	14.61	0.72	0.86	< 0.001	0.78
SCC^3^	1.81	1.96	0.10	0.32	0.002	0.61
ECM^4^	32.23	30.99	0.68	0.26	< 0.001	0.15
FCM^5^	33.39	31.93	0.87	0.29	0.01	0.14
ECM/DMI	2.86	2.78	0.07	0.42	< 0.001	0.20
FCM/DMI	2.96	2.86	0.08	0.45	0.003	0.20

^1^During Phase 1, intake and MYs were measured daily, BW and BCS once per week and milk composition two times a week. During Phase 2, intake, MYs and milk composition were measured daily, BW and BCS at the start and end of the phase. There were eight cows per treatment.

^2^MUN = milk urea nitrogen.

^3^SCC = somatic cell count. Somatic cell count data were log-transformed and results are presented in the log scale.

^4^Energy corrected milk = [12.82 × fat yield (kg)] + [7.13 × protein yield (kg)] + [0.323 × MY (kg)] (Hutjens, 2010).

^5^Fat corrected milk = (0.4324 × kg of MY) + (16.216 × kg of milk fat) (Hutjens, 2010).

There was an overall TRT effect for MY during the P2 FR challenge, with CON cows having greater MY than NTK cows (*P* = 0.04; Figure 1). Lactose yield was also lower in NTK cows during phase 2 (*P* = 0.02), while the yield of SNF tended to be lower in NTK vs. CON cows (*P* = 0.09). No TRT × Day interactions were observed for performance parameters during P2 (*P* > 0.10; Table 2). Day effects were observed for all production parameters during the P2 FR challenge (*P* < 0.05), except for DMI and fat yield (*P* > 0.10). Dry matter intake, MY, BW, BCS, EB, protein percentage and yield, lactose percentage and yield, total solids percentage yield, ECM, FCM, ECM/DMI, and FCM/DMI all decreased across FR, while fat percentage, MUN, and SCC increased (Figures 2 and 3 and Supplemental Figures S1–4)

### Plasma biomarkers

During P1 no TRT effects observed for the concentrations of plasma biomarkers of energy metabolism, inflammation, oxidative stress, liver function, and Mg during P1 (*P* > 0.10; Table 3). The only TRT × Day effect was a tendency (*P* = 0.08) for plasma NEFA concentrations (Figure 3), but no statistical differences between treatment groups were detected at any time point (*P* > 0.15). There were, however, concentrations of several biomarkers that varied by day during P1 (Supplemental Figure S5–8). Plasma creatinine concentrations decreased from d 30 to 63 (*P* = 0.06), while PON and cholesterol concentrations decreased across P1 (*P* = 0.04 and 0.03, respectively), concentrations of ROMt stayed the same between d 1 and 30 but were decreased on d 63 (*P* = 0.002), while FRAP decreased from d 1 to 30 but then increased again on d 63 to levels similar to d 1 (*P* = 0.03). At last, myeloperoxidase concentrations were similar on d 1 and 30, but decreased on d 63 (*P* = 0.001), while plasma ceruloplasmin tended to decrease across P1 (*P* = 0.06).

**Table 3.** Least square means and associated standard error for plasma biomarkers in Holstein cows supplemented with a *Saccharomyces cerevisiae* fermentation product (NTK) or a CON for 9 wk and subjected to a FR challenge for 5 d (40% of ad libitum intake). Data are separated according to experimental period; treatment feeding (Phase 1; 0–63 d) and FR challenge (Phase 2; 64–68)^1^

Item^2^	Treatment		SEM	*P*-value		
	CON	NTK		Trt	Day	Trt × Day
*** Phase 1***						
Energy Metabolism						
BHBA (mmol/L)	0.62	0.63	0.02	0.68	0.76	0.38
Creatinine (μmol/L)	81.05	82.11	0.96	0.46	0.06	0.24
Glucose (mmol/L)	4.76	4.77	0.05	0.85	0.13	0.53
NEFA (mmol/L)	0.12	0.12	0.01	0.90	0.002	0.08
Urea (mmol/L)	4.14	4.62	0.22	0.15	0.12	0.18
Liver function						
AST/GOT (U/L)	108	110	8.53	0.89	0.59	0.97
Cholesterol (mmol/L)	5.46	5.36	0.19	0.72	0.03	0.54
GGT (U/L)	31.24	33.71	2.68	0.54	0.13	0.98
Paraoxonase (U/mL)	96.15	96.24	8.07	0.99	0.04	0.47
Total bilirubin (μmol/L)	0.96	1.13	0.08	0.18	0.21	0.91
Inflammation						
Ceruloplasmin (μmol/L)	2.81	2.55	0.20	0.37	0.06	0.50
Haptoglobin (g/L)	0.29	0.20	0.08	0.49	0.50	0.84
Myeloperoxidase (U/L)	374	321	30.7	0.25	0.002	0.59
Oxidative stress						
FRAP (μmol/L)^6^	159.01	156.15	7.59	0.80	0.03	0.56
ROMt (mg H_2_O_2_/100 mL)	16.13	14.70	1.22	0.43	0.002	0.93
Minerals						
Mg (mmol/L)	1.09	1.09	0.02	0.94	0.41	0.62
***Phase 2***						
Energy Metabolism						
BHBA (mmol/L)	0.54	0.53	0.03	0.89	< 0.001	0.89
Creatinine (μmol/L)	81.78	84.65	1.30	0.16	0.02	0.75
Glucose (mmol/L)	4.54	4.58	0.09	0.76	0.002	0.55
NEFA (mmol/L)	0.68	0.67	0.05	0.91	< 0.001	0.97
Urea (mmol/L)	5.16	5.47	0.15	0.19	< 0.001	0.52
Liver function						
AST/GOT (U/L)	107	109	8.01	0.85	< 0.001	0.65
Cholesterol (mmol/L)	5.48	5.48	0.18	0.99	< 0.001	0.71
GGT (U/L)	33.75	36.64	2.42	0.43	< 0.001	0.80
Paraoxonase (U/mL)	89.48	90.33	7.30	0.94	0.01	0.46
Total bilirubin (μmol/L)	2.64	2.50	0.19	0.60	< 0.001	0.17
Inflammation						
Ceruloplasmin (μmol/L)	2.69	2.32	0.24	0.32	0.64	0.60
Haptoglobin (g/L)	0.17	0.18	0.02	0.87	0.19	0.07
Myeloperoxidase (U/L)	281	217	34.69	0.18	0.89	0.25
Oxidative stress						
FRAP (μmol/L)^6^	149	139	8.03	0.41	0.01	0.77
ROMt (mg H_2_O_2_/100mL)	14.22	12.71	1.40	0.47	0.73	0.57
Minerals						
Mg (mmol/L)	1.01	1.03	0.02	0.53	< 0.001	0.14

^1^ Blood was collected from the coccygeal vein ~1 h before feeding on d 1, 30, and 63 during Phase 1 and on d 64 (right before restriction started), 65, 66, 67, 68, and 69 (right before they left the experiment). There were eight cows per treatment.

^2^AST/GOT = aspartate aminotransferase; BHBA = beta hydroxybutyrate; GGT = γ-glutamyl transpeptidase; FRAP = ferric reducing ability of plasma; Mg = magnesium; NEFA = non-esterified fatty acids; ROMt = reactive oxygen species, total.

There were also no TRT effects on plasma biomarkers during the P2 FR challenge (*P* > 0.10; Table 3). However, there was a tendency for a TRT × Day interaction for plasma concentrations of haptoglobin (*P* = 0.07; Figure 3), where concentrations were greater in CON cows on the second day of FR (NTK: 0.13 g/L, CON: 0.26 g/L, SEM: ± 0.03 g/L; *P* = 0.01). Several effects of Day were also observed for plasma biomarkers during the P2 FR challenge (Supplemental Figures S5–8). Plasma NEFA concentrations increased during the first 3 d of FR and then plateaued for the rest of the phase (*P* < 0.001). Concentrations of BHBA were also affected by Day (*P* < 0.001), decreasing from d 1 to 2 and then increasing to a peak on d 5 of FR. Plasma glucose also changed over Day (*P* = 0.002); concentrations decreased through d 4 and then increased slightly. A Day effect was also observed for creatinine (*P* = 0.02) with concentrations increasing from d 1 to 2, but then remained elevated. In addition, urea concentrations increased sharply from d 1 to 2, but then decreased through d 4 before rising again (*P* < 0.001). Plasma concentrations of AST/GOT, cholesterol and bilirubin all increased during the first 3 d of FR, but then decreased (*P* < 0.001 for all). Concentrations of PON decreased steadily across the FR challenge (*P* = 0.01), while GGT concentrations increased (*P* < 0.001). Of the minerals and markers of oxidative stress, Mg (*P* < 0.001), and FRAP (*P* = 0.01) decreased during the FR challenge.

### Immune function

**Figure 4.**
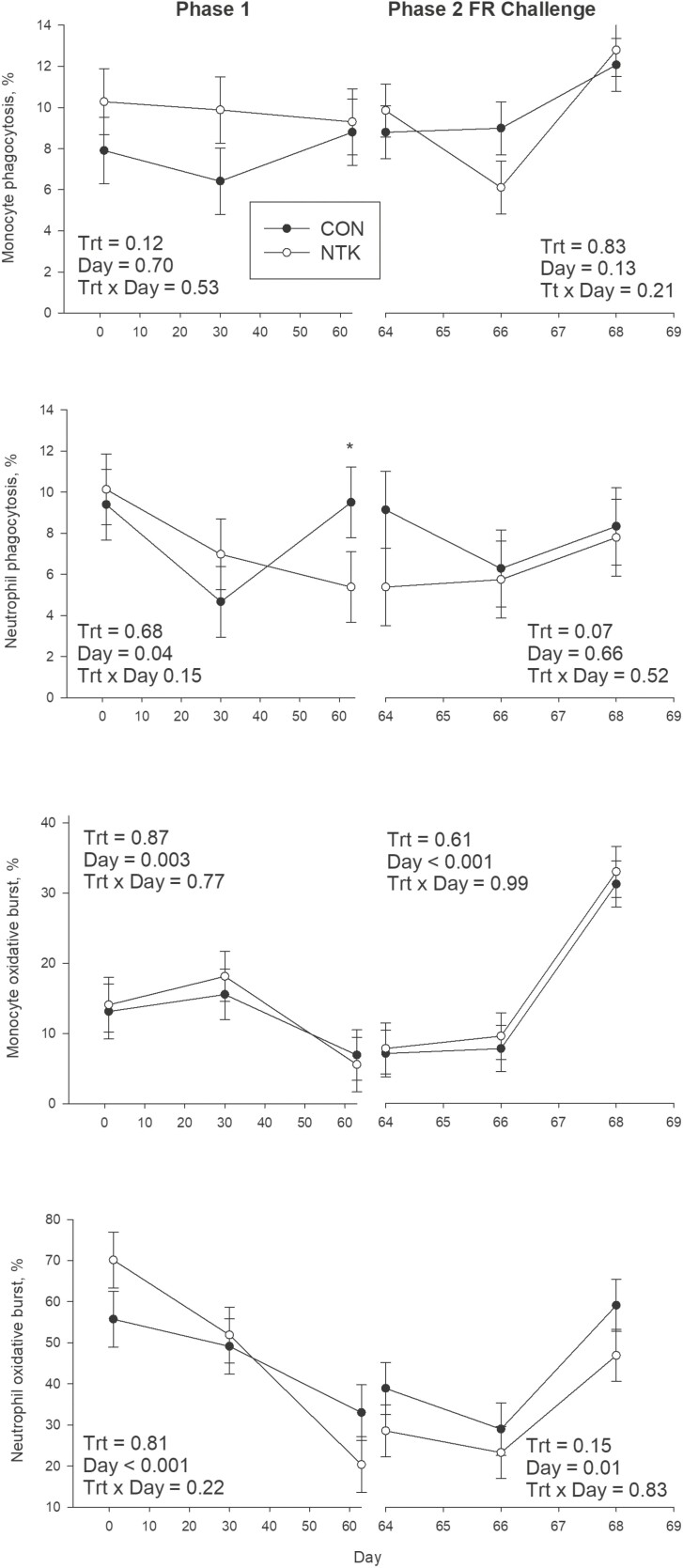
Least square means and associated standard error for neutrophil and monocyte phagocytosis and oxidative burst in Holstein cows supplemented with a *Saccharomyces cerevisiae* fermentation product (NTK) or a CON for 9 wk and subjected to a FR challenge for 5 d (40% of ad libitum intake). Data are separated according to experimental period; treatment feeding (Phase 1; 0–63 d) and FR challenge (Phase 2; d 64–68). Blood was collected on ~1 h prior to feeding from the coccygeal vein on d 1, 30, and 63 in Phase 1 and on d 64, 66, and 68 during Phase 2. There were eight cows per treatment. *P ≤ 0.05.

No TRT effects were observed during P1 for neutrophil phagocytosis (*P* = 0.68; Figure 4) or oxidative burst (*P* = 0.81). While monocyte oxidative burst was not different between CON and NTK cows during P1 (0.87), there was a tendency for monocyte phagocytosis to be greater in NTK cows (NTK: 9.82%, CON: 7.71%, SEM: ± 0.86%; *P* = 0.12). There was a tendency for only one TRT × Day interaction (*P* = 0.15; Figure 1), where CON cows had lower neutrophil phagocytosis on d 63. In addition, both neutrophil and monocyte oxidative burst capacity decreased over time during P1 (*P <* 0.001 and *P* = 0.003, respectively; Figure 2).

No TRT × Day interactions were observed for any immune function parameters in the FR period (*P* > 0.10; Figure 4). During FR, there was a tendency for neutrophil phagocytosis to be lower in NTK cows than CON (NTK: 5.40%, CON 8.04%, SEM ± 0.07; *P* = 0.07), as well as a tendency for neutrophil oxidative burst to be lower in NTK cows than CON (NTK: 26.20%, CON: 36.93%, SEM: ± 5.15%; *P* = 0.15). There were no differences in monocyte phagocytosis (*P* = 0.83) or oxidative burst (*P* = 0.61); however, several effects of Day on immune function were observed during FR. Neutrophil oxidative burst decreased slightly and then increased at the end of the 5 d FR challenge (*P* = 0.01), while there was a tendency for monocyte phagocytosis to change in the same way across Day (*P* = 0.13). At last, monocyte oxidative burst capacity increased 4-fold from the start until the end of the FR challenge (7.24% to 31.33%).

## Discussion

In the present study, cows were exposed to an FR challenge intake to induce disruptions in immunometabolism and intestinal barrier function. An FR challenge at 40% of ad libitum intake previously induced disruptions in intestinal barrier function (Kvidera et al., 2017b) and was chosen as the model for the present study. Although milk production decreased during the FR challenge regardless of diet, changes in plasma biomarkers of inflammation were not significantly altered.

### Production parameters

The increases in DMI, ECM, FCM, milk fat, and protein in the first 9 wk, i.e., P1, support previous observations summarized in a comprehensive meta-analysis by Poppy et al. (2012). Thus, production results from P1 in the present experiment provide further support for the beneficial effects of SCFP on milk and milk components throughout lactation. It is worth noting however, that Poppy et al. (2012) observed an increase in MY that drove increases in fat and protein yields, which contrasts with the increase in fat and protein percentages, but not MY in the present study. A mechanism behind the large increases in milk fat and protein in the present study is not clear; however, one factor could be the greater DMI of the NTK cows compared with CON. In addition, SCFP supplementation has previously been associated with improvements in epithelial tissue barrier (Vailati-Riboni et al., 2021). It is also possible that NTK cows may have had better intestinal barrier function, which would have allowed for better absorption of nutrients. At last, SCFP supplementation has been associated with improved ruminal fermentation (Hristov et al., 2010), suggesting that another factor contributing to the milk component responses observed in the present study could be better ruminal fermentation in NTK cow vs. CON. Such a response, however, was not evaluated in the present study.

The lack of effect during the P2 FR challenge on DMI, ECM, or FCM, while opposite to P1 and the results of the meta-analysis by Poppy et al. (2012), is similar to previous studies where cows were fed SCFP during an immunologic or metabolic challenge. For instance, during a mastitis challenge with *Streptococcus uberis*, Vailati-Riboni et al. (2021) did not observe differences in production parameters with SCFP supplementation, either during the first 45 d feeding period, or during the 3-wk period of recovery from the challenge. However, during the challenge, there was an interaction between treatment and time, indicating a better recovery from the challenge in SCFP cows compared with controls (Vailati-Riboni et al., 2021). Furthermore, no differences in DMI, MY, or milk components were observed when SCFP was fed to cows during a 7 d severe abrupt heat stress challenge using electric heat blankets (Al-Qaisi et al., 2020).

The fact that cows receiving NTK had lower MY during the FR in the present study was somewhat surprising. This change in MY also drove the observed decrease in lactose yield since there was no difference in lactose percentage between treatments. Such a decrease in MY and lactose yield has not been observed previously with SCFP supplementation (Poppy et al., 2012). It is possible that changes in lactose and milk production during FR were associated with the greater DMI and ECM in P1; increases in intake and production are known to increase organ size, particularly of the gastrointestinal tract, in ruminants (Ortigues and Doreau, 1995), resulting in greater maintenance costs for those tissues. Thus, it could be speculated that during FR, the NTK cows were experiencing greater maintenance costs during FR due to changes in the organ size in P1. As such, compared with CON cows, the nutrient supply (including glucose) to the mammary gland for milk production in FR could have been reduced. Further work is needed to fully elucidate the mechanisms by which SCFP might alter lactose yield during a period of abrupt FR.

### Plasma biomarkers

The lack of effect of NTK on plasma concentrations of biomarkers of inflammation, oxidative stress, metabolism, or liver function during either phase of the present experiment was not wholly unexpected based on previous studies feeding SCFP. For instance, Vailati-Riboni et al. (2021) did not observe changes in metabolites such as glucose and NEFA, acute-phase proteins such as haptoglobin and albumin, reactive oxygen metabolites or cytokines when cows were fed SCFP and challenged with a mastitis pathogen. Al-Qaisi et al. (2020) also did not observe differences in plasma glucose, NEFA, BHBA, or insulin with SCFP supplementation during a period of heat stress. Furthermore, during the periparturient period Olagaray et al. (2019) did not observe differences in metabolic biomarkers, and Knoblock et al. (2019) did not observe differences in markers of oxidative stress or serum amyloid A, but did observe lower concentrations of haptoglobin on d 7 after calving in SCFP cows compared with controls. Thus, the lack of effects of NTK on plasma biomarkers in the present study are in line with previous immunometabolic challenges and suggest that NTK supplementation has minimal effects on a broad range of systemic biomarkers during periods of abrupt FR.

As is typical of a period of FR or decreased DMI, concentrations of NEFA and BHBA increased during P2. However, it is noteworthy that at a systemic level, concentrations of biomarkers of inflammation and oxidative stress were not greatly increased over time during FR. This was somewhat surprising since we know that periods of reduced DMI, such as the periparturient period, are associated with increases in inflammation and oxidative stress (Loor et al., 2013a, 2013b). In addition, in previous work restricting cows to the same level of FR used here (40% ad libitum intake) Kvidera et al. (2017b) observed increases in markers of inflammation, including haptoglobin, endotoxin, lipopolysaccharide-binding protein, and serum amyloid A. However, it is possible that the lack of changes in plasma biomarkers associated with inflammation and oxidative stress was partly due to the design of our experiment. While our FR was severe at 40% of ad libitum intake, the length was relatively short. During a longer period of decreased feed intake, such as the periparturient period (3 wk pre- and postpartum), cows experience immense physiological and immunological changes (Loor et al., 2013a, 2013b). Thus, our 5 d FR challenge may not have been long enough to elicit major changes in inflammation and oxidative stress at a systemic level.

Pascottini et al. (2019) restricted transition cows to 60% of their average intake for 4 consecutive d (d −15 to −12 precalving) and did not observe differences in markers of systemic inflammation such as haptoglobin or lipopolysaccharide-binding protein. Further support for this hypothesis comes from previous work where restricting cows to 60% of net energy for lactation requirements for 4 d also did not significantly alter plasma concentrations of inflammation and oxidative stress biomarkers (Coleman et al., 2019). In addition, cows in the present study were past peak lactation (>60 DIM) and were not experiencing any sort of immunologic or metabolic challenge, and, thus, not experiencing inflammation or oxidative stress. This circumstance may also explain the lack of changes in biomarkers of inflammation and oxidative stress during the P2 FR challenge.

### Immune function

*Saccharomyces cerevisiae* fermentation products are known to contain a variety of bioactive compounds such as beta glucans, B vitamins, nucleotides, and amino acids (Hristov et al., 2010) that have been linked to activation of the immune system (Li et al., 2005). In dairy cattle specifically, research over the last few years has begun to link improvements in health and production with SCFP and its bioactive compounds activating immune responses and thereby priming the immune system (Al-Qaisi et al., 2020; Mahmoud et al., 2020; Vailati-Riboni et al., 2021). Thus, we hypothesized that supplementation with an SCFP in the present study would improve the innate immune response as measured via an ex vivo assay during a period of abrupt FR. Although during P1, there was a weak tendency for monocyte phagocytosis to be greater with NTK, suggesting a better monocyte response, contrary to our hypothesis, neutrophil phagocytosis tended to be lower in NTK cows during the P2 FR challenge, while neutrophil oxidative burst had a weak tendency to be lower in NTK cows. It should be noted that the tendency for an interaction of treatment and day for neutrophil phagocytosis during P1 indicated that CON cows had greater neutrophil phagocytosis at the end of P1, which suggests they experienced an event that caused an increase in phagocytosis at that point when it had been decreasing as lactation progressed. However, there were no unusual health events recorded around the end of P1 to explain such an increase in phagocytosis.

Previously, in heat-stressed cows fed SCFP there was an increase in circulating white blood cells and neutrophils (Al-Qaisi et al., 2020). In addition, while feeding SCFP did not alter phagocytosis or oxidative burst of neutrophils or monocytes in calves challenged with bovine respiratory syncytial virus, immune cells from SCFP calves produced less proinflammatory cytokines and had better mucosal immune responses in the lungs resulting in reduced lung tissue damage (Mahmoud et al., 2020). Similarly, during a mastitis ­challenge, cows on a control diet (CON) experienced increased activity of both neutrophils and monocytes 30 h postinfection, while cows supplemented with SCFP remained constant (Vailati-Riboni et al., 2021). In the same study, they also observed better immune cell responses/activity and barrier function (assessed via gene expression analysis) at the level of the mammary gland in SCFP cows compared with the CON (Vailati-Riboni et al., 2021). Taking into account the effects in both the mammary gland and circulation, those authors speculated that control cows may have been experiencing leakage of bacterial components into circulation, leading to activation before they reached the infected mammary tissue (Vailati-Riboni et al., 2021).

Although leakage of intestinal components to the circulation was not assessed, we speculate that the tendency for lower neutrophil function during FR may be due to priming of the immune system and mucosal barriers with NTK compared with CON. Periods of FR are associated with increased passage of microbes and endotoxins into circulation (Kvidera et al., 2017a). Thus, it could be speculated that cows receiving NTK in the present study were experiencing less passage of toxins into circulation during FR, decreasing the need for an innate immune response, i.e., NTK cows were better prepared and needed less of an immune response during FR than CON cows. However, circulating concentrations of toxins were not measured in the present study to confirm this hypothesis.

## Conclusions

Supplementation of SCFP for 9 wk confirmed its positive effects on milk production via increases in milk fat, protein, ECM, FCM, and feed efficiency. However, during a period of abrupt FR (40% of ad libitum intake) for 5 d, SCFP supplementation reduced yields of milk and lactose and did not alter plasma biomarkers of inflammation, oxidative stress, metabolism, or liver function. FR for 5 d also did not significantly increase systemic markers of inflammation and oxidative stress. Further work is needed to understand the possible effects of NTK on mucosal barrier function during periods of stress.

## Supplementary Material

skad019_suppl_Supplementary_Material

## Abbreviations

AST: aspartate aminotransferase
BCS: body condition score
BHB: β-hydroxybutyrate
CON: total mixed ration with no supplementation
DIM: days in milk
ECM: energy-corrected milk
FCM: fat-corrected milk
FR: feed restriction
FRAP: ferric reducing ability of plasma
GGT: γ-glutamyl transpeptidase
MPO: myeloperoxidase
MUN: milk urea nitrogen
MY: milk yield
NEFAs: non-esterified fatty acids
NRC: National research council
NTK: total mixed ration supplemented with NutriTek®
PON: paraoxonase
ROM: total plasma reactive oxygen metabolites
SCC: somatic cell count
SCFP: *Saccharomyces cerevisiae* fermentation
SNF: solids non-fat
TMR: total mixed ration
