# Supplementary material for: Feeding a Saccharomyces cerevisiae fermentation product before and during a feed restriction challenge on milk production, plasma biomarkers, and immune function in Holstein cows
Source: J Anim Sci. 2023 Jan 14;101:skad019. doi: 10.1093/jas/skad019 (PMC9992451; doi:10.1093/jas/skad019)
Supplement: skad019_suppl_Supplementary_Material [file skad019_suppl_supplementary_material.docx]

**Table S1.** Initial parameters (average ± s.d.) at enrollment before treatment assignment.

| **Parameter^1^** | **CON** | **NTK** |
| --- | --- | --- |
| n | 8 | 8 |
| Lactation | 3.4 ± 0.74 | 3.5 ± 0.53 |
| DIM | 98.1 ± 7 | 96.1 ± 8 |
| DMI (kg/d) | 26.80 ± 2.68 | 27.71 ± 3.00 |
| Milk^2^ (kg/d) | 42.2 ± 6.24 | 43.8 ± 7.13 |
| BW^3^ | 765 ± 49 | 742 ± 37 |
| BCS^3^ | 2.99 ± 0.39 | 2.86 ± 0.22 |
| SCC^4^ | 59 ± 35 | 51 ± 40 |

^1^CON = control; NTK = *saccharomyces cerevisiae* fermentation product supplemented group; BW = body weight; BCS = body condition score; DIM = days in milk; DMI = dry matter intake; SCC = somatic cell count.

^2^Average of data collected during the adaptation period

^3^One time measurement collected during the adaptation period (1 wk before treatment allocation).

^4^Average of the two DHI tests conducted before enrollment.

**Supplemental Figure S1.** Least square means and associated standard error for body condition score, body weight and energy balance of Holstein cows supplemented with a *Saccharomyces cerevisiae* fermentation product (NTK) or a control diet (CON) for 9 wk and subjected to a feed restriction (FR) challenge for 5 d (40% of ad libitum intake). Data are separated according to experimental period; treatment feeding (Phase 1; 0-63 d) and FR challenge (Phase 2; d 64-68). There were 8 cows per treatment.

**Supplemental Figure S2.** Least square means and associated standard error for energy-corrected milk (ECM) and fat-corrected milk (FCM) of Holstein cows supplemented with a *Saccharomyces cerevisiae* fermentation product (NTK) or a control diet (CON) for 9 wk and subjected to a feed restriction (FR) challenge for 5 d (40% of ad libitum intake). Data are separated according to experimental period; treatment feeding (Phase 1; 0-63 d) and FR challenge (Phase 2; d 64-68). There were 8 cows per treatment. * represents significant difference at *P* ≤ 0.05 and + a tendency at *P* ≤ 0.10.

**Supplemental Figure S3.** Least square means and associated standard error for feed efficiency as calculated by energy-corrected milk/DMI and fat-corrected milk/DMI of Holstein cows supplemented with a *Saccharomyces cerevisiae* fermentation product (NTK) or a control diet (CON) for 9 wk and subjected to a feed restriction (FR) challenge for 5 d (40% of ad libitum intake). Data are separated according to experimental period; treatment feeding (Phase 1; 0-63 d) and FR challenge (Phase 2; d 64-68). There were 8 cows per treatment. * represents significant difference at *P* ≤ 0.05 and + a tendency at *P* ≤ 0.10

**Supplemental Figure S4.** Least square means and associated standard error for lactose percentage and yield, milk urea nitrogen (MUN) and somatic cell count (SCC) of Holstein cows supplemented with a *Saccharomyces cerevisiae* fermentation product (NTK) or a control diet (CON) for 9 wk and subjected to a feed restriction (FR) challenge for 5 d (40% of ad libitum intake). Data are separated according to experimental period; treatment feeding (Phase 1; 0-63 d) and FR challenge (Phase 2; d 64-68). There were 8 cows per treatment. * represents significant difference at *P* ≤ 0.05 and + a tendency at *P* ≤ 0.10.

**Figure S5.** Least square means and associated standard errors for creatine, glucose, urea and beta-hydroxybutyrate in Holstein cows supplemented with a *Saccharomyces cerevisiae* fermentation product (NTK) or a control diet (CON) for 9 wk and subjected to a feed restriction (FR) challenge for 5 d (40% of ad libitum intake). Data are separated according to experimental period; treatment feeding (Phase 1; 0-63 d) and FR challenge (Phase 2; d 64-68). Blood was collected on ~1 h prior to feeding from the coccygeal vein on d 1, 30 and 63 in phase 1 and daily during phase 2. There were 8 cows per treatment. * represents significant difference at *P* ≤ 0.05 and + a tendency at *P* ≤ 0.10.

**Figure S6.** Least square means and associated standard errors for plasma myeloperoxidase and ceruloplasmin in Holstein cows supplemented with a *Saccharomyces cerevisiae* fermentation product (NTK) or a control diet (CON) for 9 wk and subjected to a feed restriction (FR) challenge for 5 d (40% of ad libitum intake). Data are separated according to experimental period; treatment feeding (Phase 1; 0-63 d) and FR challenge (Phase 2; d 64-68). Blood was collected on ~1 h prior to feeding from the coccygeal vein on d 1, 30 and 63 in phase 1 and daily during phase 2. There were 8 cows per treatment. * represents significant difference at *P* ≤ 0.05 and + a tendency at *P* ≤ 0.10.

**Figure S7.** Least square means and associated standard errors for plasma aspartate aminotransferase, paraoxonase, cholesterol, γ-glutamyl transpeptidase and bilirubin in Holstein cows supplemented with a *Saccharomyces cerevisiae* fermentation product (NTK) or a control diet (CON) for 9 wk. in Holstein cows supplemented with a *Saccharomyces cerevisiae* fermentation product (NutriTek, Diamond V, Cedar Rapids, IA: NTK) or a control diet (CON) for 9 wk and subjected to a feed restriction (FR) challenge for 5 d (40% of ad libitum intake). Data are separated according to experimental period; treatment feeding (Phase 1; 0-63 d) and FR challenge (Phase 2; d 64-68). Blood was collected on ~1 h prior to feeding from the coccygeal vein on d 1, 30 and 63 in phase 1 and daily during phase 2. There were 8 cows per treatment. * represents significant difference at *P* ≤ 0.05 and + a tendency at *P* ≤ 0.10.

**Figure S8.** Least square means and associated standard errors for plasma reactive oxygen metabolites total, ferric reducing ability of plasma and magnesium in Holstein cows supplemented with a *Saccharomyces cerevisiae* fermentation product (NTK) or a control diet (CON) for 9 wk and subjected to a feed restriction (FR) challenge for 5 d (40% of ad libitum intake). Data are separated according to experimental period; treatment feeding (Phase 1; 0-63 d) and FR challenge (Phase 2; d 64-68). Blood was collected on ~1 h prior to feeding from the coccygeal vein on d 1, 30 and 63 in phase 1 and daily during phase 2. There were 8 cows per treatment. * represents significant difference at *P* ≤ 0.05 and + a tendency at *P* ≤ 0.10.
